# First person – Tobias Beigl, Ine Kjosås and Emilie Seljeseth

**DOI:** 10.1242/bio.057497

**Published:** 2020-12-02

## Abstract

First Person is a series of interviews with the first authors of a selection of papers published in Biology Open, helping early-career researchers promote themselves alongside their papers. Tobias Beigl, Ine Kjosås and Emilie Seljeseth are co-first authors on ‘Efficient and crucial quality control of HAP1 cell ploidy status’, published in BiO. Tobias is a PhD student in the laboratory of Professor Walter E. Aulitzky at the Dr Margarete Fischer-Bosch Institute for Clinical Pharmacology, Stuttgart, investigating protein biology from the N- to the C-terminus and back again. Ine is a Master's student in the laboratory of Professor Thomas Arnesen at the University of Bergen, Institute of Biological Science. Emilie is a Master's student in the laboratory of Nadra J. Nilsen at NTNU Department of Clinical and Molecular Medicine in Trondheim, investigating the dual role of immune cells in cancer.


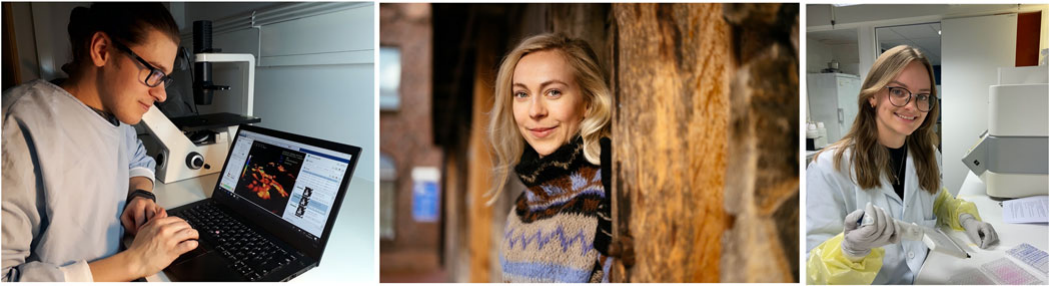


**Tobias Beigl, Ine Kjosås and Emilie Seljeseth.**

**What is your scientific background and the general focus of your lab?**

The general interest of the Arnesen lab focusses on fundamental questions related to the biology of protein modifications. More precisely, it provides pioneering work on the field of N-terminal acetylation of proteins. This modification is carried out by a specific enzyme family called N-terminal acetyltransferases (NATs). N-terminal acetylation occurs in a multitude of proteins in eukaryotes crucially influencing protein properties and function in manifold cases. Hence, we made it our mission to investigate its influence on cellular processes, unravel connections to human disease and get to the bottom of NAT biology.

**How would you explain the main findings of your paper to non-scientific family and friends?**

Science is not possible without very specific and reliable tools. One of these tools are, e.g. commercially available and genetically engineered cell lines like the HAP1 cell line described in our article. Scientists all over the world use these cells to generate sensitive data with a potentially far-reaching impact. In our publication, we highlight an intriguing phenomenon of the HAP1 cell line: the cells, originally containing only a single copy of their DNA, can double their DNA spontaneously. We showed that this could influence crucial aspects of their biology and thus potentially the results obtained with HAP1 cells. However, our work presents an effective methodology to counter this problem, isolate the cells with the same amount of genetic material and hence ensuring robust and comparable data acquisition using HAP1 cells as a scientific tool.

“…we highlight an intriguing phenomenon of the HAP1 cell line: the cells, originally containing only a single copy of their DNA, can double their DNA spontaneously.”

**What are the potential implications of these results for your field of research?**

The presented method to quality-control the ploidy status of HAP1 cells is not only important for our field but all fields of research using HAP1 cells in experiments. The ploidy instability of the HAP1 cells could lead to a third variable problem. We have showed that ploidy status is a vital factor for many cellular features. We hope our findings can raise awareness of the scientific society towards this need for quality control. Our results render analysis of HAP1 diploidy status not only important but also necessary before working with these cells. In the long term, the here presented streamlined workflow may become a part of the usual handling protocol for the HAP1 cell line.

**What has surprised you the most while conducting your research?**

For us, it was most fascinating, that a crucial phenomenon like the spontaneous diploidization of HAP1 cells has not been targeted before. Also, that there has not been a uniform procedure yet despite the fact that these cells are made commercially available on such a big scale. Our group became aware of the ploidy instability factor when we purchased the cells for phenotypic analyses. We think that many scientists have to face this surprise as well or are unaware and therefore at risk of making comparisons between wild-type and knockout cells of different ploidy state. We have worked out a promising approach for an easy and effective solution.

**What, in your opinion, are some of the greatest achievements in your field and how has this influenced your research?**

Talking about the HAP1 cells as a tool, the biggest achievement here is the vast number of different CRISPR/Cas9-edited clones that are commercially available by now. The CRISPR/Cas technology is undoubtedly one of the greater achievements of biological and biochemical science of the 21st century as acknowledged with the recent Nobel prize. Combined with haploid cell lines like HAP1, gene-editing companies like Horizon Discovery gained a powerful tool to generate clones with virtually any genetic modification desired. With over 7500 different edited cell lines available by now, scientists can now acquire these tools in much less time and with much less effort. For the research in our lab, knockout cell lines of HAP1 cells made a big contribution to recent studies about crucial functions of the NAT proteins.

**Figure:**
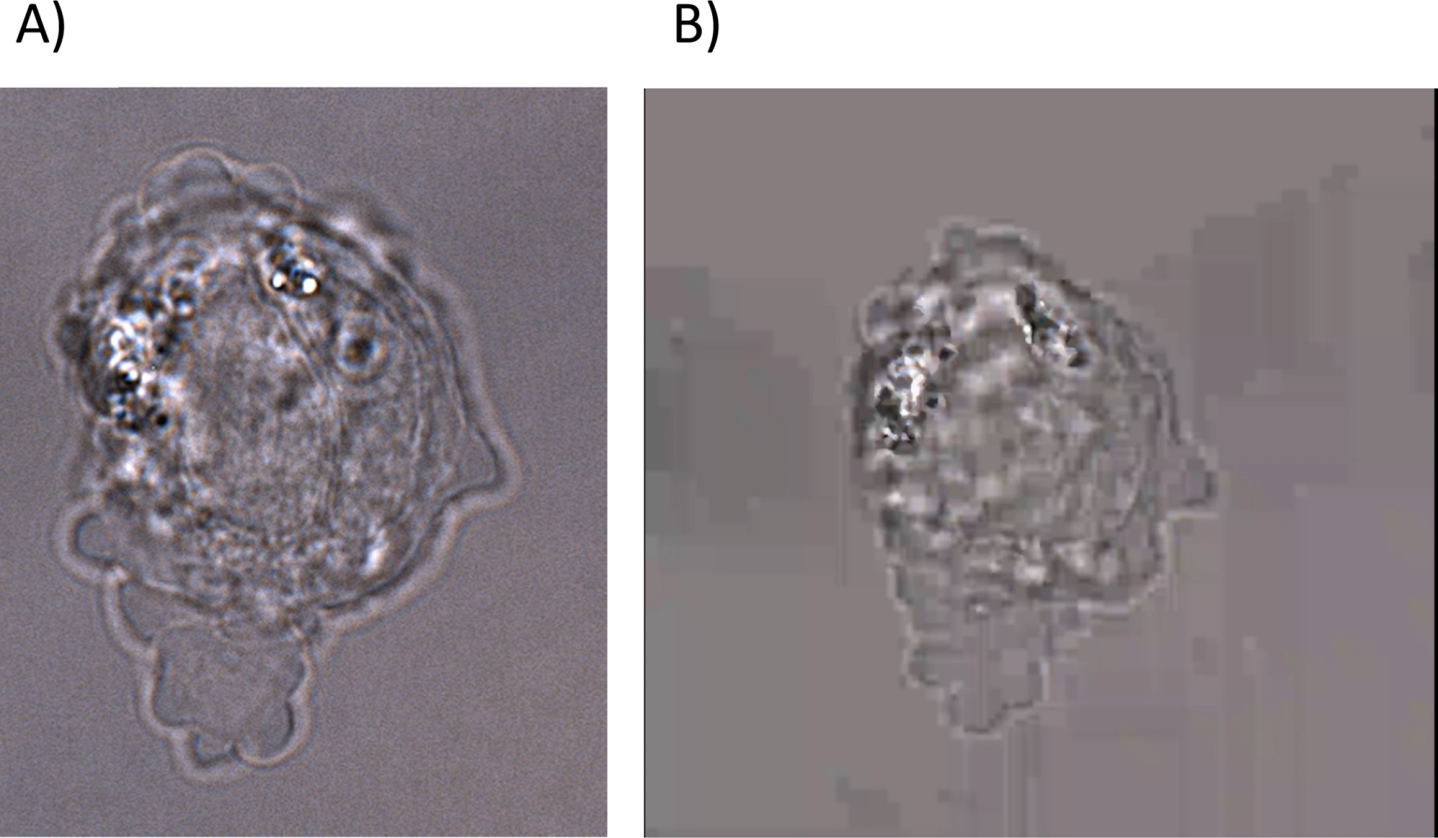
**Phenotypic assay of a gene specific HAP1 Knockout cell line A: image, B: still from Movie 1.** The cell line was verified as diploid using our streamlined protocol for ploidy quality control prior to live cell imaging. Images were obtained by phase-contrast microscopy using a Nikon TE2000 wide-field microscope, 60× oil-lense with 1.5 magnification. Data were processed using Nikon NIS Element Viewer. The video that B is taken from is a 10-min time-lapse compressed to 10s and can be found in the supplementary material file.

**What changes do you think could improve the professional lives of early-career scientists?**

First and foremost, it comes down to first-class support by the supervisor or PI in the lab. One should be encouraged early to get active, ask questions, participate in discussions, and be involved in important processes like the writing of a manuscript. Furthermore, among the scientific society, especially among high-ranking scientists and journals, young researchers should be recognized, e.g. at conferences where PhD and even Master's students get opportunities to be in the spotlight. There should be an atmosphere without contempt and disdain, where it is clearly conveyed at all levels that making mistakes is okay and that you are in the process of learning. Also, there is no doubt that a higher number of financial support programs are necessary, not to mention adequate payment, e.g. for PhD students.

“There should be an atmosphere without contempt and disdain, where it is clearly conveyed at all levels that making mistakes is okay…”

**What's next for you?**

**T.B.:** After finishing my Master's in the Arnesen lab in which I contributed to the current publication, I decided to start a PhD in my home country, Germany. Therefore, I switched fields in the meantime, from N-terminal acetylation and dealing with HAP1 knockout cell lines to investigating certain domains of the Bcl-2 family proteins and dealing with resistance mechanisms in cancer cell lines. In this new scientific environment as well, I hope to continue my career with further findings, conferences and publications during my PhD.

**I.K.:** I am writing my Master's thesis at the Arnesen lab, studying a novel NAT. Hopefully, the future will bring greater insight to the role of protein-modification and its impact on human physiology. Further, I would like to take part in an evolving scientific environment, if possible as a PhD, to get a greater understanding of the functions and mechanisms in our cells.

**E.S.:** I recently started my Master's project at the Centre of Molecular Inflammation Research (CEMIR), NTNU. Through my work at CEMIR, I hope to uncover new insights about the dual role of the immune system in cancer, with an emphasis on how different subsets of neutrophils can affect cancer progression. I hope to add to my toolbox of experimental techniques and continuously work on my scientific thinking.

## Supplementary Material

Supplementary information

## References

[BIO057497C1] Beigl T. B., Kjosås I., Seljeseth E., Glomnes N. and Aksnes H. (2020). Efficient and crucial quality control of HAP1 cell ploidy status. *Biology Open* 9, bio057174 10.1242/bio.057174 33184093 PMC7673356

