# Supplementary material for: First person – Tobias Beigl, Ine Kjosås and Emilie Seljeseth
Source: Biol Open. 2020 Dec 2;9(11):bio057497. doi: 10.1242/bio.057497 (PMC7725594; doi:10.1242/bio.057497)
Supplement: Supplementary information [file biolopen-9-057497-s1.pdf]

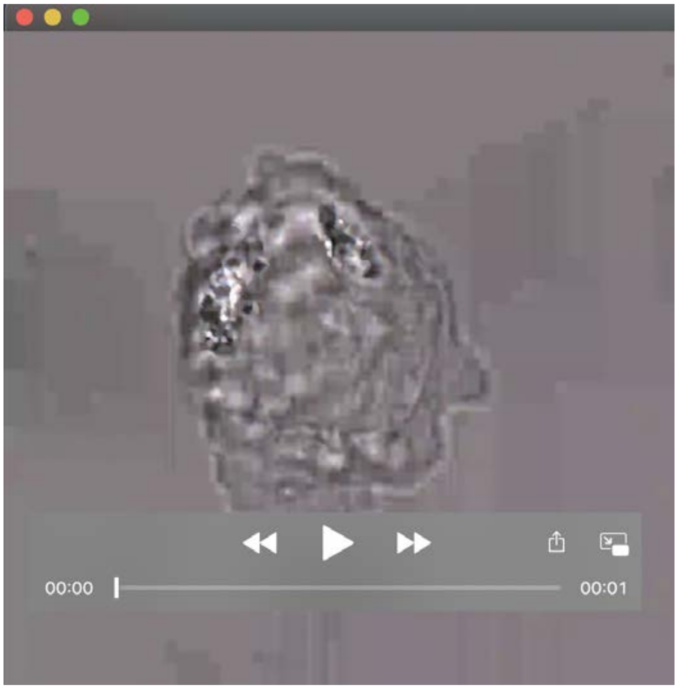

Movie 1. Phenotypic assay of a gene specific HAP1 Knock-out cell line. The cell line was verified as diploid using our streamlined protocol for ploidy quality control prior to live cell imaging. Data were processed using Nikon NIS Element Viewer. The video is a 10-min time-lapse compressed to 10s.

Biology Open • Supplementary information
